# Expression of dual-specificity phosphatases in TGFβ1-induced EMT in SKOV3 cells

**DOI:** 10.55730/1300-0144.5626

**Published:** 2023-02-21

**Authors:** Sabire GÜLER, Abdullah YALÇIN

**Affiliations:** 1 Department of Histology and Embryology, Faculty of Veterinary Medicine, Bursa Uludag University, Bursa, Turkey; 2 Department of Biochemistry, Faculty of Veterinary Medicine, Bursa Uludag University, Bursa, Turkey

**Keywords:** Dual-specificity phosphatases, epithelial-mesenchymal transition, TGFβ1, ovarian carcinoma, SKOV3, MAPK

## Abstract

**Background/aim:**

The study aims to profile the dual-specificity phosphatases (DUSP) expression in response to Transforming growth factor β1 (TGFβ1)-induced epithelial-mesenchymal transition (EMT) in ovarian adenocarcinoma cells.

**Materials and methods:**

The ovarian adenocarcinoma cell line SKOV3 was used as a TGFβ1-induced EMT model. Cells were incubated with 5 ng/mL TGFβ1 to induce EMT. EMT was confirmed with real-time qPCR, western blot, and immunofluorescence analyses of various EMT markers. Western blot was used to analyze phospho- and total MAPK protein levels. Typical and atypical DUSPs mRNA expression profile was determined by real-time qPCR.

**Results:**

The epithelial marker E-cadherin expressions were decreased and mesenchymal EMT markers Snail and Slug expression levels were increased after TGFβ1 induction. Phosphorylation of ERK1/2 and p38 MAPK were enhanced in response to TGFβ1 treatment. The expression of DUSP2, DUSP6, DUSP8, DUSP10, and DUSP13 were decreased while DUSP7, DUSP16, DUSP18, DUSP21, and DUSP27 were increased by TGFβ1.

**Conclusion:**

TGFβ1 induced EMT which was accompanied by increased activity of MAPKs, and led to marked changes in expressions of several DUSPs in SKOV3 cells.

## 1. Introduction

Epithelial ovarian cancer is the most lethal gynecological cancer with 46% survival rate after diagnosis, owing to metastatic progression and recurrence [1,2]. Serous ovarian carcinoma is the most frequent histological subtype of ovarian carcinoma [3]. In carcinoma cells, the EMT (epithelial-mesenchymal transition) program is directly associated with malignant features such as invasion, metastasis, and resistance to chemotherapy [4,5]. TGFβ1 (Transforming growth factor β1) has a key role in the induction of EMT [6]. TGFβ activates EMT through SMAD-dependent and SMAD-independent pathways, which are mediated by the phosphorylation and activation of SMAD and MAPK (mitogen-activated protein kinases) proteins, respectively [7,8]. Next, suppression of the epithelial gene (E-cadherin) and upregulation of mesenchymal genes (N-cadherin and Fibronectin) and transcription factors (Snail, Slug, and ZEB1) initiate the EMT [9,10]. As a result, cells lose their epithelial morphologies, and acquire mesenchymal traits that are associated with enhanced malignant properties [11].

Clarifying the molecules associated with the TGFβ1 signaling pathway, whose role has been proven in the initiation and maintenance of EMT, is critical for identifying new drug targets. Studies reveal enzymes that control the phosphorylation of proteins involved in the regulation of intracellular signaling pathways initiated by molecules such as TGFβ1 as potential target molecules [12]. Of these, DUSPs are recognized as key regulators of MAPK proteins. DUSPs comprise typical and atypical DUSPs, depending on whether they can contain a MAPK-interacting domain (also known as kinase interaction motif; KIM) [13]. The expression of DUSPs and their roles in the development of tumor progression and drug resistance have been evaluated in several tumor types, including ovarian cancer [14,15]. DUSPs have overlapping and nonoverlapping functions. While expression of some DUSPs is associated with malignant properties, expression of others has been shown to correlate with patient survival. For example, in high-grade serous ovarian cancer, DUSP1 expression was found to be associated with worse survival in serous ovarian cancer [16]. Down-regulation of DUSP2 expression in serous ovarian carcinoma was found to be associated with poor survival, and its expression in SKOV3 and OVCAR3 cells inhibited cell proliferation and migration in vitro [15]. Given the importance of MAPK activity in the progression of various cancers, including ovarian cancer, the profiling of MAPK-regulating DUSPs in a context and stimuli-dependent manner may reveal distinct DUSPs whose activities can be rationally modulated for the treatment of cancer [17].

In ovarian cancer progress and metastasis, TGFβ1 is known to induce EMT, which is accompanied by increased MAPK activity [18,19]. Although a limited number of DUSP expressions have been studied in ovarian cancer, regulation of DUSP expression by TGFβ1 in serous ovarian carcinoma cells is currently unknown. In the present study, we aimed to profile all known DUSPs after TGFβ1 induction in the serous ovarian adenocarcinomas cell line SKOV3.

## 2. Materials and methods

### 2.1. Cell culture and treatment

SKOV3 (ATCC, HTB-77™) cells were cultured in 10% fetal bovine serum (Gibco, 10270106)-supplemented McCoy’s 5a Modified Medium (Biological Industries, 01-075-1A). The cells were grown at 37 °C in 5% CO_2_. The recombinant TGFβ1 PeproTech (100-21C) was used at 5 ng/mL concentration.

### 2.2. Immunofluorescence

Cells were cultured on coverslips, and fixed with 4% formaldehyde. The recommended protocol of antibody manufacturer (Cell Signaling) was followed. The cells were incubated with the primary antibodies E-cadherin (Cell Signaling, 3195), Slug (Cell Signaling, 9585), fibronectin (Sigma, F7387), vimentin (Cell Signaling, 5741) at 4 °C overnight and probed with Alexa-Fluor 488-conjugated goat antirabbit antibody (Cell Signaling, 4412) for fluorescence detection. The images were visualized under EVOS Imaging System (ThermoFischer, U.S.A.).

### 2.3. Real-time quantitative PCR

RNA isolation (ThermoFischer, K0732) and cDNA synthesis (ThermoFischer, 4368814) were carried out following the manufacturer’s instructions. Real-time quantitative PCR (qPCR) analyses were performed on StepOne Plus (ThermoFischer, U.S.A) using gene expression master mix (Promega, A6101) and gene-specific TaqMan probes (ThermoFisher). The probes used were Snail (Hs00195591_m1), Slug (Hs00161904_m1), E-cadherin (4331182 Hs01023895_m1), N-cadherin (Hs01023894_m1), fibronectin (Hs01549976_m1), ZEB1 (Hs00232783_m1), DUSP1 (Hs00610256_g1), DUSP2 (Hs01091226_g1), DUSP3 (Hs01115776_m1), DUSP4 (Hs01027785_m1), DUSP5 (Hs00244839_m1), DUSP6 (Hs04329643_s1), DUSP7 (Hs00997002_m1), DUSP8 (Hs00792712_g1), DUSP9 (Hs01046584_g1), DUSP10 (Hs00200527_m1), DUSP11 (Hs01061375_m1), DUSP12 (Hs00170898_m1), DUSP13 (Hs00969203_m1), DUSP14 (Hs01877076_s1), DUSP15 (Hs01566654_m1), DUSP16 (Hs00411837_m1), DUSP18 (Hs01036622_g1), DUSP19 (Hs00369901_m1), DUSP21 (Hs00254403_s1), DUSP22 (Hs00414885_m1), DUSP23 (Hs00367783_m1), DUSP26 (Hs00225167_m1), DUSP27 (Hs01367756_m1), DUSP28 (Hs01374134_m1), and GAPDH (Hs03929097_g1).

### 2.4. Western blot analysis

Western blot analysis was performed as previously described [20]. Briefly, cells were washed twice in cold PBS and lysed with lysis buffer for setting up protein lysates. The proteins were resolved by 4%–12% SDS-PAGE gel electrophoresis and transblotted onto polyvinylidene fluoride membrane. After blocking with 5% nonfat dry milk in Tris-buffered saline, the membranes were incubated with primer antibodies [Snail (Cell Signaling, 3879), Slug (Cell Signaling, 9585), E-cadherin (Cell Signaling, 3195), ERK1/2 (Cell Signaling, 4695), phospho-ERK1/2 (Cell Signaling, 4370), JNK (Cell Signaling, 9252), phospho-JNK (Cell Signaling, 4668), p38 (Cell Signaling, 8690), phospho-p38 (Cell Signaling, 4511), α-Tubulin (Cell Signalling, 2144), and β-actin (Cell Signaling, 3700)] overnight at 4 °C. HRP-conjugated goat antirabbit (Cell Signaling, 7074) or antimouse (Cell Signaling, 7076) secondary antibodies were used. Immunoreactive bands were visualized by using chemiluminescence (Luminata Forte HRP Substrate, Millipore) in ChemiDoc™ MP Imaging System (Bio-Rad).

### 2.5. Statistical analyses

Experiments were performed three times. Data were expressed as the mean ± SD of triplicate measurements of a single experiment, except for mRNA levels which were done in duplicate replicates. Two-sided and paired t-test was used to assess a statistical significance. p < 0.05 was considered significant.

## 3. Results

**Figure 1:**
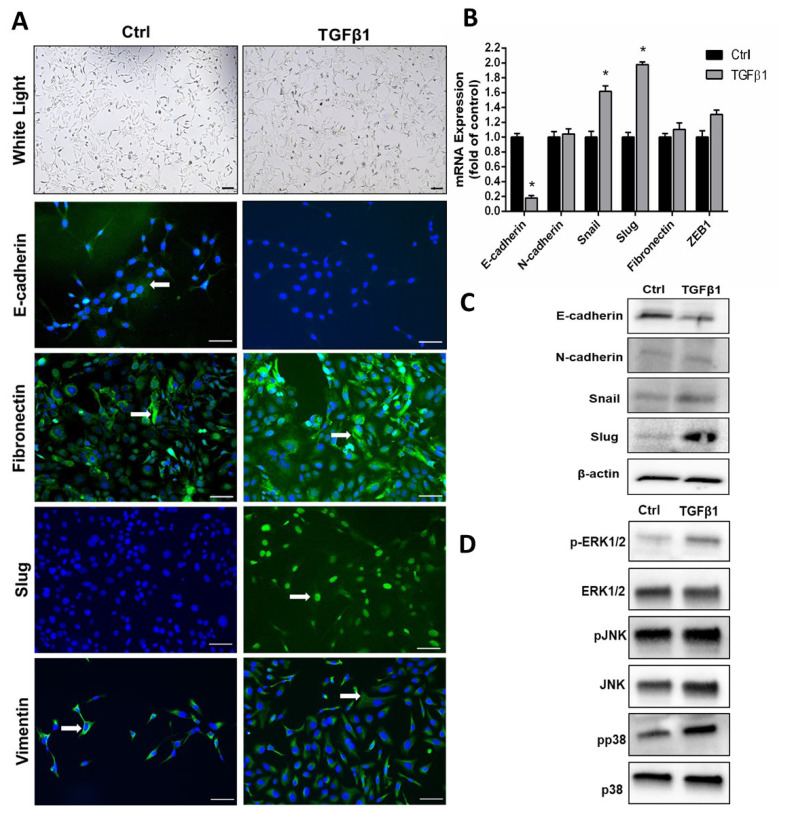
TGFβ1 induces EMT and activates MAPK pathway in SKOV3 cells. **A)** Morphology of cells and immunofluorescence analyses of epithelial and mesenchymal markers (green, arrows). DAPI was used for nuclear staining (blue). **B)** Real-time qPCR analyses. **C)** Western blot analyses of EMT markers. **D)** Western blot analyses of phosphorylated and total levels of MAPK proteins (JNK, ERK1/2, and p38 MAPK) in control and TGFβ1-treated cells. Data are presented as the mean ± SD of three replicates. Ctrl, Control. ∗p < 0.05, compared to Ctrl.

SKOV3 cells were incubated with 5 ng/mL TGFβ1 for 48 h. The cells were then examined in light microscope for morphological changes. As expected, TGFβ1 addition led to changes characterized by acquisition of fibroblast-like mesenchymal appearance (Figure 1A). To confirm EMT, expressions of epithelial and mesenchymal markers were investigated with further analyses. We observed a near complete disappearance of E-cadherin expression upon TGFβ1 administration by immunofluorescence analysis (Figure 1A). On the contrary, Slug expression, which was not observed in the control cells, markedly increased in TGFβ1-treated cells (Figure 1A). Fibronectin and vimentin staining intensities were stronger in TGFβ1-added cells (Figure 1A). We then performed real-time qPCR analyses on EMT markers. As seen in Figure 1B, TGFβ1 significantly suppressed E-cadherin mRNA levels, and induced Snail and Slug mRNA levels. Consistently, Western blot analyses revealed a decrease in E-cadherin expression and an increase in Snail and Slug expressions (Figure 1C). Confirmation of EMT was performed in all three analyses by showing the decrease in epithelial marker E-cadherin and the significant increase in mesenchymal marker Slug after TGFβ1 induction. However, after TGFβ1 induction, increase in both mRNA and protein levels of the mesenchymal marker Snail has been shown. It has been proven that EMT occurs after TGF induction with these findings and the change in vimentin localization. Then we investigated total- and phospho-protein levels of ERK1/2, JNK, and p38 to examine the effect of TGFβ1 on MAPK pathway activity in SKOV3 cells. TGFβ1 supplementation increased the phosphorylated forms of ERK1/2 and p38 (Figure 1D).

**Figure 2:**
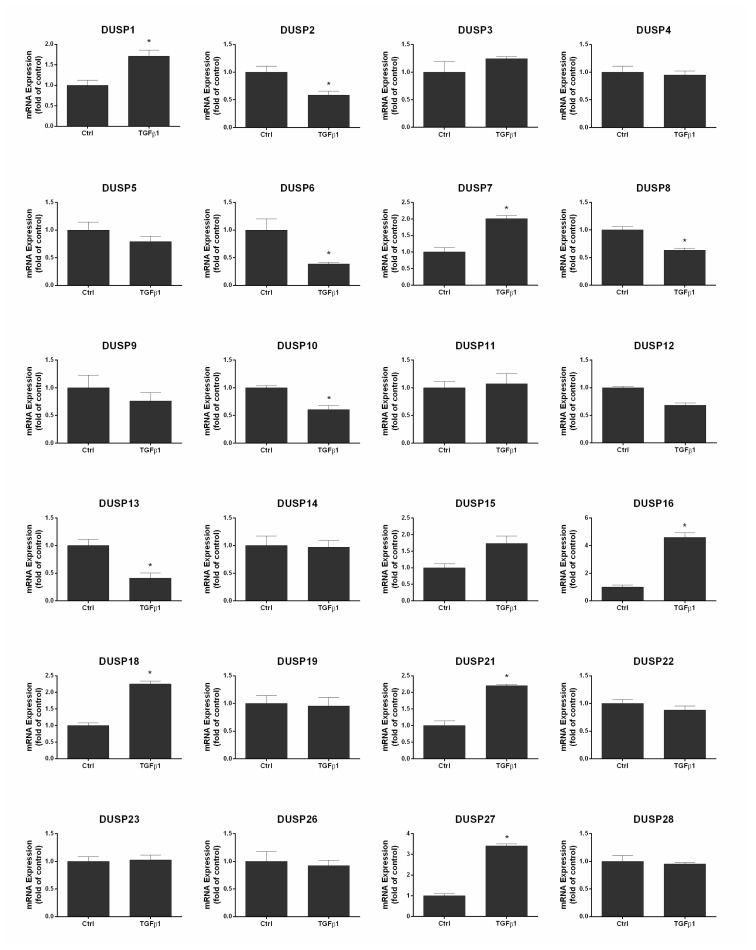
TGFβ1 causes changes in the expression of DUSPs. Real-time qPCR analyses for typical and atypical DUSPs in SKOV3 cells. Ctrl, Control. ∗p < 0.05, compared to Ctrl.

To determine the effect of TGFβ1 on expressions of all the known typical and atypical DUSP genes, mRNA expressions were examined with real-time qPCR. The expression of DUSP2, DUSP6, DUSP8, DUSP10, and DUSP13 were decreased while DUSP7, DUSP16, DUSP18, DUSP21, and DUSP27 were increased after TGFβ1 supplementation (Figure 2).

## 4. Discussion

DUSP proteins are recognized as key regulators of MAPK proteins which play essential roles in TGFβ1-induced EMT, which is associated with the acquisition and maintenance of malignant features and chemotherapy resistance in ovarian cancer [21]. Although several DUSP proteins have been associated with malignant properties of ovarian cancer cells, whether TGFβ1 causes changes in DUSP expressions is currently unknown [22]. In this study, we aim to determine the profile of all known DUSPs after induction of EMT by TGFβ1 in the SKOV3 cell line.

In SKOV3 cells, EMT could be successfully induced by 5 ng/mL TGFβ1 as assessed by morphological changes by microscopy, and decreased E-cadherin expression and increased Snail and Slug expressions by real-time qPCR. Immunofluorescence confirmed loss of E-cadherin expression, and increased Slug expression in TGFβ1-treated cells. Localization of vimentin appeared diffuse in the cytoplasm in TGFβ1-treated cells, compared with more perinuclear localization in control cells. Our results indicating the induction of EMT in SKOV3 cells upon TGFβ1 addition are consistent with the study carried out by Hou et al. [23].

In addition to the canonical pathway involving SMAD proteins that mediate the effect of TGFβ1 in promoting EMT, activation of MAPK family proteins has also been reported to be required for full induction of the EMT by TGFβ1 in tumor cells [8]. In our study, it was shown that significant phosphorylation of ERK1/2 and p38 MAPK occurred after TGFβ1 treatment. The study by Fu et al. indicates that p38 MAPK inhibition suppresses TGFβ signal transduction in the human ovarian cancer cell line CaOV3 [24].

Due to its interactions with MAPK, some DUSP members have been shown to exhibit tumor suppressor function in different types of ovarian cancer, due to their interaction with MAPK proteins, ERK in particular [25]. The role of DUSPs in carcinogenesis has been the subject of study in many different cancers in recent years. In a previous study in our laboratory, changes in many different DUSP proteins were observed after the induction of nonsmall cell lung cancer cells (A549) with TGFβ1, and a significant decrease in DUSP4 and DUSP13 was observed. In the same study, the ectopic coexpression of DUSP4/13 suppresses TGFβ1-induced ERK1/2 phosphorylation and protein levels of the EMT transcription factors Snail and Slug proteins. Moreover, DUSP4/13 coexpression partially inhibited TGFβ1-promoted migration, invasion, and chemoresistance in A549 cells [26]. In TGF-induced pancreatic adenocarcinoma (PANC-1) cells, silencing of DUSP26 expression by siRNA markedly suppressed the effect of TGFβ1 on E-cadherin and mesenchymal genes in the cells [27]. Our study reveals that all the known typical and tested atypical DUSPs are expressed at various levels in the ovarian cancer cell line SKOV3 and TGFβ1 treatment leads to marked changes in the expression of several DUSPs. Sanders demonstrated that DUSP1 inhibition significantly inhibited tumor progression [16]. Also realizing the role of DUSP1 in regulating autophagy suggests that suppression of DUSP1 may enhance the therapeutic activity of rapamycin in ovarian cancer [28]. Similarly, in our study, DUSP1 expression increased after TGFβ1 administration. Microarray analysis identified the downregulation of DUSP4, a known inhibitor of ERK, in serous ovarian carcinomas, in contrast to ovarian serous borderline tumors, suggesting a potential tool in ovarian cancer diagnosis and patient management [29,30]. In our study, there was no significant change in DUSP4 expression upon TGFβ1 supplementation. Lim et al. showed that DUSP7 and 8 are subject to methylation-dependent silencing in epithelial ovarian cancer and can be used as clinical markers [22]. In the current study, TGFβ1 supplementation increased DUSP7 and reduced DUSP8 expression in SKOV3 cells. Inhibition of DUSP6 promotes chemosensitivity via regulation of ERK signaling in two different ovarian cancer cell lines (SKOV3 and OVCAR8 cells) [25]. In the current study, DUSP6 expression was decreased upon TGFβ1 supplementation. Our unbiased profiling revealed decreases in DUSP10 and DUSP13 expressions and increases in DUSP16, DUSP18, DUSP21, and DUSP27 expressions, which have not been reported in previous studies. Although DUSPs dephosphorylate MAPKs, some DUSPs are also known targets of MAPKs, suggesting reciprocal regulation. For example, Wang et al. showed that knockdown of ERK2 decreases MKP-1 (DUSP1) phosphorylation and induction, leading to increased cisplatin sensitivity in human ovarian cancer cell lines [17]. Whether observed changes in the above DUSP expressions in our study are secondary to changes in the activities of MAPKs proteins remains to be determined. Previous studies focused on the potential use of DUSP1, DUSP4, and DUSP6 as diagnostic markers in ovarian cancer. To our knowledge, this study is the first to examine the changes in expressions of DUSP genes upon TGFβ1-induced EMT in an ovarian carcinoma model. In addition, the increase in phosphorylation of p38 MAPK, a target kinase of DUSPs, in SKOV3 cells was shown for the first time following TGFβ1 induction. However, this study has some limitations. First, a potential requirement for the observed changes in TGFβ1-induced EMT in the current model is yet to be studied. Second, whether changes in mRNA levels of DUSP genes in response to TGFβ1 are also reflected in protein levels remains to be determined. Third, given the observation that some DUSPs are induced and some are repressed with TGFβ1, it will be crucial to study the net effect of DUSP activity in the control of MAPK signaling within the context of individual models before reaching a firm conclusion with regard to the role of DUSPs in TGFβ1-regulated EMT that is associated with MAPK activity.

In conclusion, we demonstrated that TGFβ1 activates ERK1/2 and p38 MAPK and induces in SKOV3 cells. Importantly, activation of TGFβ1 signaling is associated with marked changes in expressions of DUSPs genes in SKOV3 cells.
